# Phytochemicals Determination, and Antioxidant, Antimicrobial, Anti-Inflammatory and Anticancer Activities of Blackberry Fruits

**DOI:** 10.3390/foods12071505

**Published:** 2023-04-03

**Authors:** Lidia Gil-Martínez, Nuria Mut-Salud, José Antonio Ruiz-García, Ana Falcón-Piñeiro, Mònica Maijó-Ferré, Alberto Baños, José Manuel De la Torre-Ramírez, Enrique Guillamón, Vito Verardo, Ana María Gómez-Caravaca

**Affiliations:** 1 Department of Analytical Chemistry, University of Granada, Avda Fuentenueva, 18071 Granada, Spain; 2 Department of Microbiology, University of Granada, Avda Fuentenueva, 18071 Granada, Spain; 3 Department of Nutrition and Food Science, University of Granada, Campus of Cartuja, 18071 Granada, Spain; 4 Leitat Technological Center, Carrer Innovació, 2, 08225 Terrassa, Spain; 5 Department of Organic and Inorganic Chemistry, University of Jaen, 27071 Jaen, Spain; 6 Department of Chemical Engineering, University of Granada, Avda Fuentenueva, 18071 Granada, Spain; 7 Institute of Nutrition and Food Technology ‘José Mataix’, Biomedical Research Centre, University of Granada, Avd. Conocimiento s/n, 18100 Granada, Spain

**Keywords:** *Rubus fruticosus*, phenolic compounds, terpenic compounds, biological activity, HPLC-MS

## Abstract

A comprehensive characterization of the phytochemicals present in a blackberry fruit extract by HPLC-TOF-MS has been carried out. The main compounds in the extract were ursane-type terpenoids which, along with phenolic compounds, may be responsible for the bioactivity of the extract. In vitro antioxidant capacity was assessed through Folin–Ciocalteu (31.05 ± 4.9 mg GAE/g d.w.), FRAP (637.8 ± 3.2 μmol Fe^2+^/g d.w.), DPPH (IC_50_ 97.1 ± 2.4 μg d.w./mL) and TEAC (576.6 ± 8.3 μmol TE/g d.w.) assays. Furthermore, the extract exerted remarkable effects on in vitro cellular antioxidant activity in HUVEC cells at a concentration of 5 mg/mL. Antimicrobial activity of the extract was also tested. Most sensible microorganisms were Gram-positive bacteria, such as *E. faecalis*, *B. cereus* and Gram-negative *E. coli* (MBC of 12.5 mg/mL). IC_50_ values against colon tumoral cells HT-29 (4.9 ± 0.2 mg/mL), T-84 (5.9 ± 0.3 mg/mL) and SW-837 (5.9 ± 0.2 mg/mL) were also obtained. Furthermore, blackberry extract demonstrated anti-inflammatory activity inhibiting the secretion of pro-inflammatory IL-8 cytokines in two cellular models (HT-29 and T-84) in a concentration-dependent manner. These results support that blackberry fruits are an interesting source of bioactive compounds that may be useful in the prevention and treatment of different diseases, mainly related to oxidative stress.

## 1. Introduction

Blackberry is one of the most widespread berries of the *Rubus* genus (*Rubus fruticosus*) inside the Rosaceae family. The fruit is composed by an aggregate of droplets of 1–3 cm of diameter that change the fruit’s color from green to red to black as it ripens.

Blackberry fruits have been consumed for centuries thanks to their pleasant taste [1]. Furthermore, in recent years, berries have been proposed as a “superfood”, as they are an essential source of bioactive molecules such as vitamins A and C, carotenoids, sterols, terpenoids and phenolic compounds, with high bioactivity potential, and very low calorific content [2,3,4]. In the last few years, the identification of the bioactive molecules from blackberry fruits has been explored by various authors. The most abundant compounds characterized in blackberry extracts have been phenolic compounds, mainly anthocyanins [5], flavonols, ellagitannins [6,7] and phenolic acids [8]. Traditionally, the bioactivity of blackberry extracts has been directly related with its content in phenolic compounds. Phenolic compounds are one of the most studied groups of phytochemicals due to their broad distribution in nature and biological activity. Their high diversity in terms of chemical structure makes them suitable to interact with molecules such as enzymes, scavenge free radicals in oxidative processes or chelate metals, having the potential to modulate and participate in a wide range of biological processes [9]. The color of the blackberry fruit is determined by the presence of anthocyanins (anthocyanidin glycosides) which are the main group of flavonoids in berries, and their main bioactive component [8,10]. Other phenolic compounds present in blackberries, whose bioactivity has been reported on by a large number of in vitro tests, are flavonoids and ellagitannins [10,11,12]. For example, Dragana et al. obtained a blackberry extract with a high reducing power, whose bioactivity was highly correlated with anthocyanin content [13]. On the contrary, PCA analysis carried out by Halim et al. identified phenolic acids and rutin hydrate as the main contributors of the antioxidant capacity of blackberry fruits [14]. Dai et al. also obtained a blackberry extract enriched in anthocyanins, whose anticancer activity was also related to cyanidin-based compounds [5]. Moreover, González et al. related the antibacterial activity of blackberry with its content of phenolic compounds, mainly anthocyanins [15]. Traditionally, the antioxidant, anti-inflammatory, antidiabetic and antimicrobial activities of blackberry fruits and extracts have been attributed to phenolic compounds [16,17]. In addition, they have demonstrated benefits relating to the prevention of oxidative stress, cancer and cardiovascular diseases such as coronary heart disease and stroke, among others [2,16].

Less studied, but no less important, are terpenoid derivatives. Terpenoids are, together with phenolic compounds, one of the most abundant and diverse secondary metabolites family found in nature with diverse structures and bioactivities. They have demonstrated effectiveness as antitumor, antibacterial, antiviral, antioxidant and immunomodulatory compounds and some of them have already been used in clinical practices [18,19].

Thus, the aim of this research is to determine the main phytochemicals of blackberry fruits. Furthermore, an initial screening of the in vitro antioxidant, antimicrobial, anti-inflammatory and cytotoxic potential of the bioactives present in blackberries, has been carried out. To our knowledge, this is the first work in which terpenoids present in blackberry fruits have been identified and quantified. Consequently, although they have contrasting bioactive potential, this is the first work to take them into account in the functional activity of blackberry fruits. Thus, these analyses could demonstrate the benefits of the consumption of this kind of extract as nutraceuticals, or even the fruits themselves, for the maintenance of health.

## 2. Materials and Methods

### 2.1. Chemicals and Reagents

Folin–Ciocalteu reagent, DPPH, ABTS, TPTZ, potassium persulfate, gallic acid, Trolox and the other standards were purchased from Sigma-Aldrich (St. Louis, MO, USA). LC-MS grade methanol and water, glacial acetic acid, iron chloride hexahydrate, sodium carbonate and ethanol for the extraction were purchased from Merck KGaA (Darmstadt, Germany). Vanillic acid (≥97%), rutin (≥95%), catechin (≥99%), chlorogenic acid (≥95%), ferulic acid (≥99 %), cyanidin-3-*O*-glucoside (Cy-3-glu) (≥98%), ascorbic acid (>99%), quercetin (≥95%) and geraniol (≥98.5%) standards were also purchased from Sigma-Aldrich (St. Louis, MO, USA).

### 2.2. Plant Material

*Rubus fruticosus* fruits were collected from Pradonegro, Granada (Spain) (37°19′20″ N 3°27′10″ O). They were dried at 45 °C until a constant weight was achieved. Then, they were ground and stored at −20 °C before the extraction. 

### 2.3. Extraction Procedure

Blackberry extracts were prepared by mixing the ground dry material with a solution of ethanol/water (50:50 *v*/*v*) in a ratio 1:20. The extraction was carried out at 50 °C in constant agitation during 14.5 h. In order to separate the extract from the residue, it was centrifuged for 10 min at 14,000 rpm. Supernatants were evaporated under vacuum at 30 °C in a rotary evaporator and the dry extract was stored at −20 °C until further analysis.

### 2.4. Cell Lines and Culture

HUVEC cell lines were obtained from Cell Applications Inc. (San Diego, CA, USA). They were cultured in Endothelial Cell Growth Medium (ECGM-2) supplemented with 10% fetal bovine serum (FBS), 100 μg/mL penicillin and 100 μg/mL streptomycin and placed into an incubator with 5% CO_2_ at 37 °C until 80% confluency was reached. Cells in the logarithmic phase were used in the assays. HT-29 human colon adenocarcinoma cell line (ATCC HTB-38), T-84 human colon carcinoma cell line (ATCC CCL-248) and SW-837 human rectum adenocarcinoma (ATCC CCL-235) were obtained from the Cell Cultures Unit of the University of Granada (Granada, Spain). Peripheral blood mononuclear cells (PBMCs) were provided by the biobank of Sistema Sanitario Público de Andalucía (SPPA) from the blood samples of healthy volunteers. Tumoral cells were cultured in darkness at 37 °C and humidified atmosphere of 5% CO_2_, with Dulbecco’s modified Eagle medium (DMEM) supplemented with 10% fetal bovine serum (FBS), 10 μL/mL penicillin-streptomycin 100× and 2 mM L-glutamine. PBMCs were cultured with RPMI-1460 medium supplemented with 10% FBS.

### 2.5. Analytical Characterization of Blackberry Extract

#### 2.5.1. HPLC-ESI-TOF-MS Analysis

For the HPLC-ESI-TOF-MS analysis, the dry extract was redissolved in methanol/water (50:50, *v*/*v*) (Merk KGaA, Darmstadt, Germany). The analysis was carried out in an ACQUITY Ultra Performance LC system (Waters Corporation, Milford, MA, USA) equipped with an online vacuum degasser, an autosampler, a binary pump and a thermostatted column compartment. The separation was achieved by an ACQUITY UPLC BEH Shield RP18 column (1.7 mm, 2.1 mm × 100 mm; Waters Corporation, Milford, MA, USA) at 40 °C using the methodology described by Martín García et al. [20]. The UPLC system was coupled to an electrospray ionization (ESI) source operating in positive (for anthocyanin characterization) and negative mode, and a time-of-flight (TOF) mass detector (Waters Corporation, Milford, MA, USA).

To quantify phenolic compounds in blackberry extract, chlorogenic acid, ferulic acid, rutin, catechin and quercetin standards dissolved in methanol were used. Table S1 (Supplementary Materials) summarizes the calibration ranges and curves, regression coefficients and limits of detection and quantification (LOD and LOQ).

#### 2.5.2. Total Terpenoid Content

The determination of the total terpenoid content was carried out according to Lukowski et al.’s methodology with some modifications [21]. First, 200 μL of diluted blackberry extract in methanol (50 mg/mL), methanol (blank) or standard (10–0.1 mg/mL geraniol) were added to 1.5 mL of chloroform. The mixture was vortexed and left to rest for 3 min. Then, 100 μL of concentrated sulfuric acid (65% *v*/*v*) was added. Assay tubes were incubated for 2 h in the dark (standard solutions were incubated for 5 min in the dark) and the supernatant was removed. Then, 1.5 mL of methanol was added and absorbance was measured at 538 nm. Total terpenoid content was calculated as mg geraniol equivalents/g dry weight (mg GE/g d.w.). 

### 2.6. Antioxidant Capacity Assays

#### 2.6.1. Folin–Ciocalteu Assay

The Folin–Ciocalteu methodology described by Ainsworth et al. with some modifications, was followed to determine the total antioxidant content of the extract [22]. Briefly, 400 μL of sample, standard or 80 % methanol blank was mixed with 800 μL of 10% (*v*/*v*) Folin–Ciocalteu reagent in test tubes. Then, 3200 μL of 700 mM Na_2_CO_3_ was added and incubated at room temperature for 2 h. Finally, the absorbance was read at a λ = 765 nm. Gallic acid was used as the standard in a concentration ranging from 25 to 400 ppm. Results were expressed as mg gallic acid equivalents/g of dry weight (mg GAE/g d.w.).

#### 2.6.2. Trolox Equivalent Antioxidant Capacity (TEAC) Assay

TEAC assay was performed as previously described by Re et al. [23]. The reaction between a 7 mM solution of ABTS with 2.45 mM potassium persulfate during 16 h produced the ABTS^+^ cation which can be detected at a λ = 734 nm. The solution was adjusted until an absorbance of 1.1 (±0.02). Then, 2850 μL of ABTS^+^ solution was mixed with 150 mL of solvent (blank), standard or sample. Results were expressed as μmol Trolox equivalents/g of dry weight (μmol TE/g d.w.).

#### 2.6.3. Ferric Reducing Antioxidant Power (FRAP)

FRAP assay was carried out according to the method developed by Benzie and Strain with some modifications [24]. The FRAP reactive solution consisted of 10 volumes of a 300 mM acetate buffer (pH = 3.6), 1 volume of 10 mM acid solution of TPTZ and 1 volume of a 20 mM FeCl_3_ solution. FeSO_4_ was used as the standard. Then, 3000 μL of FRAP reactive solution was mixed with 480 μL of solvent (blank), standard or sample in triplicate and the absorbance was read at a λ = 593 nm. Results were expressed in μmol Fe^2+^ eq./g of dry extract (μmol Fe^2+^ eq./g d.w.).

#### 2.6.4. DPPH Assay

Radical scavenging activity of the extract was measured using a slightly modified version of the method reported by Brand-Williams [25]. In brief, 100 μL of solvent (blank) or sample was mixed with 3900 μL of DPPH 60 μM. Mixtures were left to stand in the dark at room temperature for 30 min, and the absorbance was read at 515 nm. The inhibition rate (I%) was calculated with the formula:

I% = [(A_DPPH_ − A_blank_) − (A_s-DPPH_ − A_s-blank_)]/(A_DPPH_ − A_blank_) × 100

where A_DPPH_ was the absorbance of the DPPH solution, A_blank_ the absorbance of methanol instead of DPPH, A_s-DPPH_ the absorbance of the DPPH solution with sample and A_s-blank_ the absorbance of methanol with sample.

The EC_50_ value was defined as the concentration of dry extract required to obtain a 50 % inhibition of DPPH radical and was calculated through a calibration curve using concentrations of the extract ranging from 0.5 to 61 μg/mL. 

### 2.7. Intracellular Antioxidant Activity Assay

Intracellular lipid peroxidation was evaluated following the method described by Dayoub et al. with some modifications [26]. In brief, confluent human endothelial cells (HUVEC) were cultured in 12-well plates and treated with the blackberry extract at different concentrations (5, 10 and 20 mg/mL) and quercetin 25 μM. After 24 h, the cell medium was changed with tert-butyl hydroperoxide (TBH) 50 μM in treated and positive control wells. Then, 24 h afterwards, wells were washed twice with cold PBS, lysed with MDA lysis + butylated hydroxytoluene buffer and frozen at −20 °C. Lysates were thawed and frozen several times to ensure cell lysate. The samples were centrifuged and the supernatant obtained was used for the subsequent analysis measuring the concentration of malondialdehyde (MDA) with a lipid peroxidation MDA assay kit (Abcam, Cambridge, UK).

### 2.8. Antimicrobial Analysis

*Salmonella enterica* CECT 7160, *Escherichia coli* CECT 405, *Shigella sonnei* CECT 457, *Pseudomonas aeruginosa* CECT 116, *Listeria monocytogenes* CECT 4032, *Staphylococcus aureus* CECT 239, *Bacillus cereus* CECT 8168, *Zygosaccharomyces bailii* CECT 11997 and *Aspergillus niger* CECT 2090 were obtained from the Spanish Collection of Type Cultures (CECT). *Enterococcus faecalis* S-47, *Candida sake* DMC03 and *Penicillium expansum* DMC01 were donated by the DMC Research pathogen collection. Mueller–Hinton broth (Scharlau, Barcelona, Spain) was used for the growth of bacteria [27] and RPMI-1640 medium with L-glutamine for yeast and fungi [28]. 

Minimum Biocidal Concentration (MBC) determination was carried out using the broth microdilution method established by the CLSI [27,28]. Decreasing concentrations (1:2) of blackberry extract from 100 to 0.78 mg/mL were prepared in the corresponding liquid culture medium. Next, they were inoculated with a 10^5^ CFU/mL microbial cell suspension. Dilutions were incubated overnight and cultured in agar plates. The lowest extract concentration that completely inhibited microbial growth was considered as the MBC.

### 2.9. In Vitro Antiproliferative Assays 

In order to calculate the IC_50_ values of the blackberry extract, cells were seeded in sterile 96-well plates (Thermo Fisher Scientific, Roskilde, Denmark) at high density (1.5 × 10^4^ cells/well) and incubated at 37 °C with 5% CO_2_ for 24 h to allow cell adhesion. Increasing concentrations of the extract ranging from 0.39 to 6.25 mg/mL were added in the corresponding wells and incubated for 48 h at 37 °C with 5% CO_2_. All the concentrations evaluated were performed in sextuplicate. The effect of the extract on tumor colorectal cell lines (HT-29, T-84 and SW-837) was evaluated using a colorimetric technique with sulforhodamine-B (SRB) as previously described by Vichai et al., 2006 [29]. Optical density values were determined by colorimetry at 490 nm using a microplate reader (Multiskan EX, Thermo Electron Corporation). The assessment of absorbance was obtained using the “SkanIt” RE 5.0 for Windows v.2.6 (Thermo Labsystems, Waltham, MA, USA) and a mathematical regression analysis for each cell line using Statgraphics software (Statistical Graphics Corp., Rockville, MD, USA) was conducted. The IC_50_ values were calculated from the semi-logarithmic dose-response curve by linear interpolation.

### 2.10. In Vitro Anti-Inflammatory Assays

In vitro anti-inflammatory assays were carried out by the method previously described by Vezza et al. [30]. First, HT-29 and T-84 cells were seeded at high density (1.5 × 10^4^ cells/well) in 96-well plates. After 24 h to achieve adhesion, supernatants of each well were discarded and different concentrations of the extract dissolved in the supplemented medium were added in the corresponding wells. After 1 h of incubation with the extract, 1 mg/mL of lipopolysaccharide from *Salmonella enterica* serotype typhimurium (LPS) was added and plates were incubated for 24 h at 37 °C and 5 % CO_2_. All the concentrations to evaluate (0.39–6.25 mg/mL) were performed in sextuplicate. After induction, supernatants were collected, centrifuged at 1000× *g* for 10 min and stored at −80 °C until cytokine determination by ELISA was performed using commercial kits following the manufacturer’s instructions (Invitrogen-Thermo Fisher Scientific). 

### 2.11. Statistical Analysis

One-way analysis of variance test (ANOVA) supplemented with Tukey’s post hoc were used for the statistical comparison of the results. Differences were considered statistically significant when *p* < 0.05. Analyses were performed using GraphPad prism 8.0 software (GraphPad Software Inc., La Jolla, CA, USA). All results were expressed as mean ± standard deviation (SD).

## 3. Results and Discussion

### 3.1. Determination of Bioactive Compounds in Blackberry Extracts by HPLC-MS

A comprehensive tentative characterization of the blackberry extract was carried out by HPLC-MS analysis by comparing the mass data with databases, commercial standards (when possible) and the literature, taking into account the experimental and calculated m/z, molecular formula and Fit Conf %. The results of phenolic compounds can be seen in Table 1.

As can be seen, 34 phenolic compounds were identified using ESI negative mode for phenolic compounds and ESI positive mode for anthocyanin detection. A total of eight anthocyanins were identified: cyanidin-*O*-glucoside (rt = 5.859), cyanidin-*O*-rutinoside (rt = 6.691), cyanidin-*O*-arabinoside (rt = 7.268), delphinidin-*O*-galactoside (rt = 11.499), cyanidin 3,5-*O*-diglucoside (rt = 11.500), delphinidin-*O*-glucoside (rt = 11.672), cyanidin-*O*-sophoroside (rt = 11.830) and a delphinidin-*O*-glucoside isomer (rt = 13.572). All of them were previously described in the blackberry [31,32]. With regard to its quantification, 2.62 mg Cy-3-glu equivalents/g d.w. were detected. Albert et al. for instance, determined 6.43 mg Cy-3-glu eq./g d.w. in ultrasound-assisted ethanolic extracts and 80% ethanol as solvent [10]. However, lower anthocyanin recovery was obtained by Siqueira dos Santos et al. with a total anthocyanin recovery of 1.39 mg Cy-3-glu/g d.w [33]. 

Apart from the mentioned compounds, nine phenolic acids and derivatives were found. A compound with molecular formula C_16_H_14_O_8_ tentatively identified as Jaboticabin eluted at 0.433 min [34]. Sinapic acid and sinapic acid hexoside were detected at retention times of 4.979 and 5.476 min, respectively. Sinapic acid was previously identified in blackberries by Canadanovic-Brunet et al., 2019. In their work, 0.15 mg/g d.w. of sinapic acid was detected in a blackberry pomace extract made with 80% ethanol [35]. In addition, ellagic acid and various derivatives were identified. Methyl gallic acid appeared at a retention time of 6.639 min. Ellagic acid pentoside and its isomer were detected at 7.707 and 7.888 min. Both were previously identified by Oszmianski et al. in wild blackberry fruits [7]. Ellagic acid appeared at a retention time of 8.726 min and it is considered the main phenolic compound present in blackberry fruits by numerous authors [7,33,36]. Nevertheless, the most abundant phenolic acid detected in this work has been ellagic acid pentoside, with a total concentration of 53.6 μg/g d.w. as the sum of its isomers. Furthermore, ellagic acid 2-rhamnoside and 3-*O*-methylellagic acid were identified at retention times of 9.276 and 9.917 min. 

It is also important to remark the presence of ellagitannins. Ellagitannins are polymers of a dimeric form of gallic acid (hexahydroxydiphenic acid) which have the ability to lactonize spontaneously to form ellagic acid [6]. Lately, they have received attention due to the high antioxidant properties that are attributed to them thanks to the high quantity of free hydroxyl groups in the molecule. Specifically, casuarictin (m/z 935 Da) was detected at a retention time of 7.519 min. Moreover, two Sanguiin H6 isomers were detected in the normal m/z working range of the instrument thanks to the identification of two peaks with m/z 934 Da, corresponding to a doubly deprotonated ion with the half of the m/z value of the parent deprotonated ion that presented a characteristic isotopic distribution of 0.5 m/z unit separation among peaks, implying that the genuine mass was twofold the observed m/z value (1870 Da) [6].

In addition, 11 flavonoids and derivatives were identified. A total of 53.84 % of the total flavonoids were quercetin derivatives such as quercetin-*O*-hexoside, quercetin-3-galactoside, quercetin-*O*-glucoside, quercetin-3-glucuronide and quercetin-*O*-acetylhexoside that eluted at retention times of 4.367, 9.046, 9.213, 9.577 and 10.199 min, respectively. Rutin and its isomer were also detected at retention times of 8.801 and 9.051 min. Three catechin derivatives, such as epicatechin and two B-type procyanidin dimers (peaks 14, 10 and 15, respectively), were also tentatively identified. All of them, were previously described in blackberry fruits [33,36,37,38]. Furthermore, at retention times of 9.984 and 10.509 min, two molecules with molecular formula C_30_H_36_O_11_ were identified as kadsurarin and its isomer, and at a retention time of 10.74 min, a compound proposed as kadsurenin B was also detected. These compounds are lignans (which were first described by Wang et al.) and, as far as we know, they have not been described before in blackberry fruits [39]. 

In addition, 31 terpenoids were characterized in the blackberry extract, which, to our knowledge, have not been determined before in blackberry (Rubus fruticosus) fruits (Table 2). Usually research studies focus on phenolic compounds and bibliography about the terpenoid content in berries is scarce. Nevertheless, it is important to note that the terpenoid family may participate substantially in the bioactivity demonstrated by blackberry fruits and extracts. Regarding terpenoids, the iridoid ebuloside (rt = 4.023 min) was identified previously on Sambucus ebulus [40], cinnamoside (rt = 5.189) in Cinnamomum cassia [41], rhodioloside A (rt = 6.117 min) in Rhodiola rosea [42], scutalpine F (rt = 9.811) in Scutelaria alpina [43] and tropeoside B1 (rt = 11.338 min) in Allium cepa [44]. Gradillas et al. identified for the first time in blackberry leaves a family of terpenoids derived from ursolic acid which we have identified in blackberry fruits and which are the most abundant family of compounds present, being 93.4% of the total terpene content and the 88.8% of the total bioactive compounds quantified in the extract. Their proposed molecular formulas were C_30_H_46_O_6_, C_30_H_46_O_7_ and C_30_H_46_O_8_, which corresponded to dihydroxyurs-12-ene-23,28-dioic acid (m/z 501) and its isomers (peaks 42, 58 and 59), trihydroxyurs-12-ene-23,28-dioic acid (also known as corosin) (m/z 517) and its isomers (peaks 52, 53 and 65) and tetrahydroxyurs-12-ene-23,28-dioic acid (m/z 533) and its isomers (peaks 48, 49 and 50), respectively [45]. The same pattern was observed with the compounds identified in peaks 47 and 51, at m/z 519 and molecular formula C_30_H_48_O_7_ (dihydroxytormentic acid and its isomer); peaks 54, 55, 56, 57 and 60 at m/z 503 and a molecular formula of C_30_H_48_O_6_ (hydroxytormentic acid and its isomer); peaks 44, 62 and 63 at m/z 487 and molecular formula C_30_H_48_O_5_ (tormentic acid and its isomers); and peaks 66 and 67 at m/z 471 and molecular formula C_30_H_48_O_4_ identified as rubitic acid and its isomer [45]. All of them are pentacyclic triterpenes, a family of compounds with contrasted bioactivity. Terpenoids have been the main family of bioactive compounds quantified (63.8 mg/g d.w.). The most abundant compounds in the extract were hydroxytormentic acid and its isomers, constituting the 29.7% of the total bioactive compounds themselves, followed by corosin and tormentic acid representing 18.9% and 12% of the total, respectively. Hydroxytormentic acid is a strong antioxidant which has demonstrated capacity to scavenge reactive oxygen species (ROS) and involvement in the regulation of antioxidant-related gene expression [46]. Furthermore, it has been shown to be neuroprotective, anti-apoptotic and anti-inflammatory [47]. Tormentic acid has also shown biological activity with anti-inflammatory, antidiabetic, antihyperlipidemic, cardioprotective, anti-cancer and antimicrobial effects, among others [48]. In general, it can be stated that pentacyclic triterpenes (and ursane derivatives in particular) have demonstrated pleiotropic activity, being useful as phytochemicals against several diseases [18,49].

Finally, we also identified other antioxidant compounds such as a glycosylated derivative of ascorbic acid, dehydroascorbic acid and dimethylcitrate at retention times 0.414, 0.433 and 1.24 min, respectively (Table 2).

### 3.2. Total Antioxidant Content (Folin–Ciocalteu) and Antioxidant Capacity (FRAP, TEAC and IC_50_) of the Extract

Total antioxidant content measured by Folin–Ciocalteu (F-C) and antioxidant activity of the extract through FRAP, DPPH and TEAC assays were performed. Quercetin and ascorbic acid were used as positive controls to compare the antioxidant activity of the extract with commercial antioxidants. Results are shown in Table 3.

It is well known that the F-C assay is not specific for the quantification of polyphenols as it interacts with other reducing nonphenolic molecules and may lead to the overestimation of the total phenolic content. In addition, it has been observed that terpenoids, such as carotenoids, can interfere in the results giving a signal two times higher than gallic acid [50]. The F-C reaction is based on electron transfer, and it can measure the reductive capacity of an antioxidant and correlates very well with other antioxidant assays used in food analysis, such as ABTS and DPPH [51]. 

The results on Folin–Ciocalteu reactive compounds (31.05 mg eq. GAE/g d.w.) were in accordance with those obtained by other researchers for blackberry extracts prepared in similar conditions with 21.43 mg GAE/g d.w. [10] and from 21.59 to 40.18 mg GAE/g d.w. for ethanolic extracts from different blackberry cultivars [52]. Dai et al. obtained lower levels of gallic acid equivalents 17.32 mg/g d.w and significantly lower antioxidant activity in terms of TEAC (66.98 μmol of TE/g d.w.) [5]. On the other hand, there are authors who have achieved superior antioxidant activity values. Santos et al., for example, prepared an extract using ultrasound-assisted extraction with 52.36 mg GAE/g d.w. measured by F-C assay but, referring to TEAC values, there were no significant differences with our extract (555.62 versus 576.6 μM TE/g d.w.) [33]. Wajs-Bonikowska et al. obtained an ethanolic extract of blackberry pomace with 94.43 mg GAE/g d.w. which also presented significantly higher antioxidant activity by ABTS (1011.22 μmol TE/g d.w.) [53]. Moreover, Zorzi et al. analyzed the antioxidant capacity through TEAC (10.25 mmol trolox/g d.w.), FRAP (7.02 mmol Fe^2+^/g d.w.) and an IC_50_ of 280 μg d.w./mL. Although TEAC and FRAP values were considerably higher, the IC_50_ value of the extract obtained by Zorzi was significantly lower than that obtained in this work [54]. 

As can be seen, there is a lot of variability in the antioxidant capacity of different blackberry extracts. Differences in those values, may be related to harvesting time, soil fertility, climatic conditions and the extraction method implemented for its obtention [10]. It is important to highlight the antioxidant effect of non-phenolic compounds, such as organic acids and their derivatives such as ascorbic acid or citric acid that may be present in the extracts, and terpenoids, which are the main compounds in the blackberry extract studied in this research [55,56,57].

Additionally, the antioxidant capacity of two commercial standards (ascorbic acid and quercetin) was measured to compare the results with those obtained for the blackberry extract. It is important to highlight that the content of bioactive compounds of the blackberry extract was 66.78 mg/g d.w. expressed as the sum of total phenolic compounds and total terpenoid content. The process of extraction is not a selective method for the obtention of bioactive compounds. Thus, during the process, sugars, fibers, proteins, fats and other compounds with low or any antioxidant activity may be extracted [58]. Even so, the antioxidant activity exerted by blackberry extract by FRAP assay was comparable to 108 mg/g d.w. of quercetin and a 73.8 mg/g d.w. of ascorbic acid. Additionally, regarding the TEAC assay, the antioxidant capacity of blackberry extract was equal to that exerted by 525.9 mg/g d.w. of quercetin and 334.6 mg/g d.w. of ascorbic acid. Thus, the bioactive compounds of blackberry extract are more efficient in the reduction process of free radicals. This may be due to the synergistic effect among the phenolic compounds and terpenoids of the extract [59,60].

### 3.3. Inhibition of Intracellular Lipid Peroxidation

**Figure 1:**
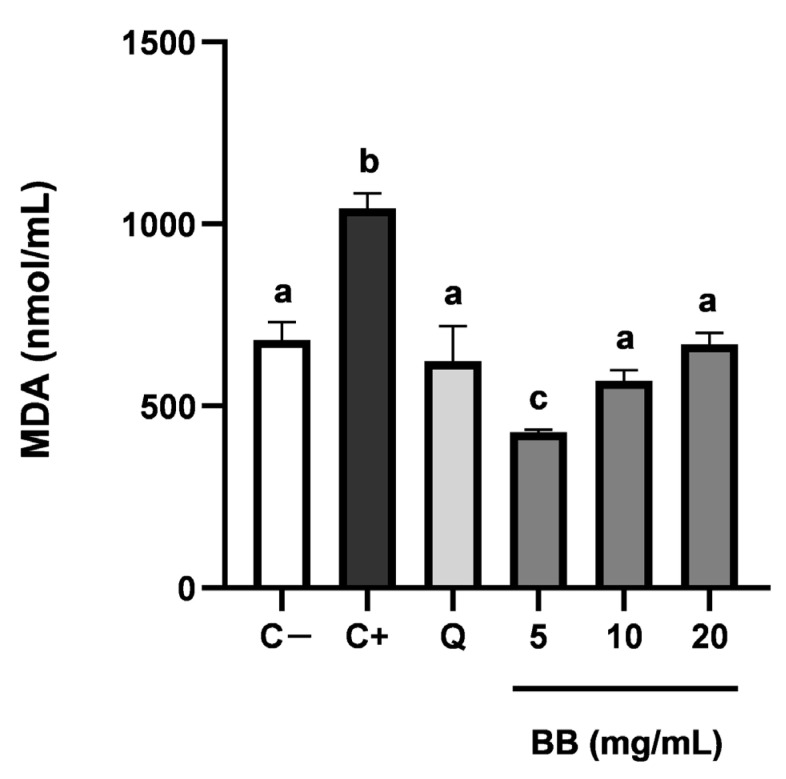
Inhibitory capacity of the blackberry extracts (BB) (5, 10 and 20 mg/mL) and quercetin 25 μM (Q) of intracellular malondialdehyde (MDA) generation as a marker of lipid oxidation induced by TBH. C− are standard HUVEC cells. C+ are HUVEC cells in contact with TBH but without treatment. Results expressed as the average nmol MDA/mL ± SD (n = 6). Groups with different letters are significantly different (*p* < 0.05).

In order to demonstrate the antioxidant activity of blackberry extract from a more biologically relevant perspective, the capacity of the extract to inhibit lipid peroxidation was tested using HUVEC cells. The results of the quantification of MDA (as an indicator of lipid peroxidation) revealed that blackberry extract prevented the intracellular lipid oxidation and the generation of MDA (Figure 1). The most effective dosage was 5 mg/mL of extract, reducing the levels of MDA significantly below the normal levels (C−) and even more than quercetin 25 μM (Q). Higher dosages such as 10 or 20 mg/mL were not as effective. This result may be due to the pro-oxidant effect that some antioxidants such as phenolic compounds may have when they are used at high concentrations [61]. Nevertheless, in our assays, it is remarkable that at higher concentrations (10 and 20 mg/mL) the blackberry extract also exerted antioxidant protection against lipid oxidation. 

Azofeifa et al. evaluated the capacity of blackberry juice to inhibit lipid peroxidation in liposomes, liver homogenates and erythrocytes demonstrating the ability of blackberry antioxidants to inhibit intracellular ROS and the formation of MDA [62]. Hassan et al. studied the in vivo capacity of blackberry ingestion to prevent oxidative stress after the administration of sodium fluoride in rats. They concluded that blackberry juice boosted the cellular antioxidant defense system preventing the damage produced by the toxicity of fluoride [63]. The same effect was observed by Cho et al. who developed a work to investigate the oxidative stress prevention in rats treated with carbon tetrachloride. They suggested that blackberry extract had significant protective activity against oxidative damage in vivo [64]. Thus, blackberry compounds have demonstrated that they are able to inhibit oxidative stress and intracellular lipid oxidation both in vitro and in vivo. Nevertheless, since not all exogenous antioxidants are effective in attenuating the oxidative stress inside the human body due to biological reasons, more studies are needed to elucidate their bioavailability, their metabolic pathways and their mechanisms of action. 

### 3.4. Antimicrobial Activity of Blackberry Extract

The antimicrobial activity of blackberry extract was tested against five Gram-positive and four Gram-negative bacteria, three yeasts and one mold. The results (MBC/MFC, mg/mL) are shown in Table 4.

In general, the most sensitive microorganisms to blackberry compounds were Gram-positive bacteria such as *E. faecalis* and *B. cereus* and the Gram-negative *E. coli*, with a MBC of 12.5 mg/mL. *L. monocytogenes*, *L. innocua*, *S. aureus* and *C. sake* were also susceptible, with a MBC of 25 mg/mL. *S. sonnei* and *Z. bailii*’s MBC were 50 mg/mL, and the rest of the Gram-negative bacteria and fungi were more resistant to the antimicrobial activity of blackberry extracts; however, at a concentration of 100 mg/mL, all microorganisms tested were killed. Radovanovic et al. studied the antimicrobial activity of wild blackberry extracts against different Gram-positive and Gram-negative bacteria. The MBC for all tested bacteria ranged from 62.5 μg/mL for *S. enteriditis* and *S. aureus* to 500 μg/mL to *E. coli*, *K. pneumoniae*, *P. vulgaris*, *C. perfringens*, *B. subtilis* and *L. inocua* [65]. On the other hand, Jazic et al. assayed the antimicrobial activity of blackberry extracts against *S. aureus*, *E. coli* and *A. niger*. In general, *S. aureus* was more susceptible than *E. coli* to blackberry extracts, and *A. niger* mycelium growth was inhibited by more than 25% at a concentration of 2.5 mg/mL [52]. In addition, Dragana et al. tested the antimicrobial activity of blackberry pomace extracts. They observed MBC values ranging from 0.78 to >25 mg/mL, with the strains of *Staphylococcus* and *P. aeruginosa* being more vulnerable to blackberry pomace extract and *C. albicans* the most resistant microorganism [13]. 

The results show that the antimicrobial activity of blackberry extracts is not always proportional to the total phenolic content [52]. Thus, this may be indicative that there are other non-phenolic molecules such as terpenoids and organic acids that might exert selective antimicrobial activity [15]. In fact, pentacyclic triterpenes similar to those identified in blackberry extract have been demonstrated as exerting antimicrobial activity against a wide range of microorganisms such as *Mycobacterium fortuitum* (MIC 1.56 μg/mL) and *Candida albicans* (MIC 12.5 μg/mL) [66]. Ursolic acid has demonstrated high antimicrobial activity against *E. faecalis*, *L. monocytogenes* and *B. cereus*, with MIC of 1.2 and 8 mg/mL, respectively [67]. Hence, it can be stated that Gram-positive bacteria are more susceptible than Gram-negative bacteria, yeast or mold to blackberry extract in general and phenolic compounds and terpenoids in particular [68]. This may be related to the presence of an outer membrane of lipopolysaccharides (LPS) in Gram-negative bacteria and the composition of the cell wall of fungi (mainly glucans, chitin and glycoproteins) because LPS and polysaccharides provide hydrophilic protection that makes the penetration of phenolic compounds and terpenoids difficult across the cell membrane to exert the antimicrobial effect [69,70].

### 3.5. Antitumoral Activity of Blackberry Extract

The antitumor activity of blackberry extract was tested against HT-29, T-84 and SW-837 cancer cell lines. The selectivity index was also calculated using PMBCs as a normal cell line (Table 5).

Blackberry extract presented antiproliferative activity against all the colorectal carcinoma cell lines tested, reducing 50% of the cell population at concentrations equal to 4.9 mg/mL in HT-29 cells and 5.9 mg/mL in T-84 and SW-837 cells. IC_50_ for PMBCs line was 108.4 mg/mL, and the blackberry extract selectivity index was 22.1 taking as reference the HT-29 cell line and 18.4 when considering the T-84 and SW-837 lines. Other authors have also reported the antiproliferative activity of blackberry extracts in colon tumor cells, indicating that blackberry phytochemicals may exert antitumor activity. For example, Seeram et al. investigated the effect of blackberry extracts in colon cancer cells HT-29 and HCT116, among other tumoral cell lines, obtaining IC_50_ values of 64.6 and 65.0 μg/mL, respectively [71]. In more recent studies, IC_50_ values of different blackberry pomace extracts tested in HT-29 cells were 505.6 to 930.6 μg/mL in the research of Jazic et al., 2019 [72] and 294 μg/mL in the investigation of Rodrigues et al., 2020 [73]. 

It is difficult to be certain that bioactive components of a natural extract are going to pass through the intestinal barrier and arrive at the target tissue without modifications in their structure. In this research work, we have focused on colorectal cancer lines, because it is more likely that bioactive components of the extract have direct contact with tumor cells inside the gastrointestinal tract and can exert their beneficial activity. Nevertheless, during the digestion process, the intestinal microbiota may modify the molecular structure of the bioactive molecules that have been characterized in this research. This might result in a modification of the in situ antitumoral performance of the extract [74]. In consequence, future research may be focused on the evaluation of the bioactivity of blackberry extract after digestion and fermentation by the intestinal microbiota. 

### 3.6. Anti-inflammatory Activity of Blackberry Extract

**Figure 2:**
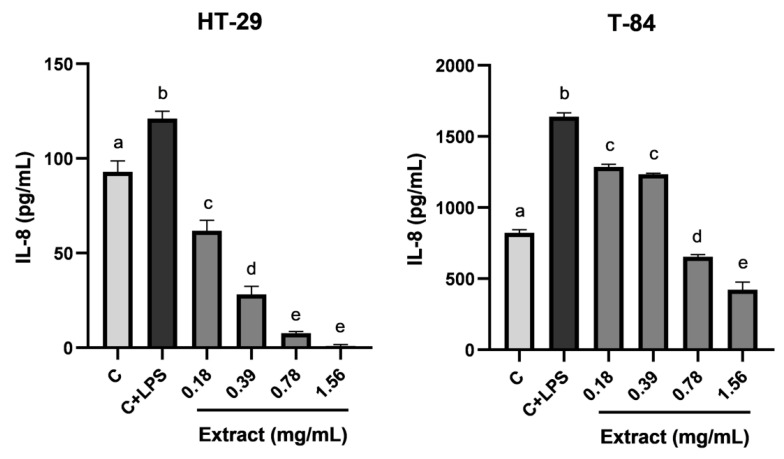
Effect of blackberry extract on levels of IL-8 produced by HT-29 and T-84 cells stimulated with LPS. Cells were induced during 24 h with LPS (1 µg/mL) and increasing concentrations of bilberry leaves extract (0.18–1.56 mg/mL). C: cells not induced with LPS (Control); C+LPS: cells induced with just LPS (1 µg/mL). The experiments were performed in triplicate. Groups with different letters are significantly different (*p* < 0.05).

To evaluate the anti-inflammatory effect of the blackberry extract, concentrations from 0.18 to 1.56 mg/mL were tested on colon tumor cells HT-29 and T-84. After the induction, the culture supernatants were analyzed to calculate the levels of pro-inflammatory cytokine IL-8 produced by tumor cells at the different conditions. As can be seen in Figure 2, there was a baseline release of IL-8 in non-stimulated cells (C) that experienced a significative increase when cells were stimulated with LPS (C+LPS). The treatment of stimulated cells with different concentrations of blackberry extract showed inhibition in the production of IL-8 in a dose-dependent manner, being the most active concentration 1.56 mg/mL in both cell lines, which almost completely inhibited the secretion of IL-8 in HT-29 cells. 

These results are in accordance with those obtained by Dai et al., who studied the reduction in pro-inflammatory IL-12 levels by blackberry extract in mice bone marrow-derived dendritic cells (DCs). IL-12 release was inhibited in a concentration-dependent manner with 37.5 μg of monomeric anthocyanins/mL of medium [5], suggesting that anthocyanins may have notorious anti-inflammatory properties. However, it is not only anthocyanins of blackberry extract that may exert anti-inflammatory activity. Other phenolic compounds and terpenoids have also demonstrated their potential in the inhibition of the inflammation process and the secretion of pro-inflammatory cytokines [17,75,76]. Therefore it might be possible that the anti-inflammatory activity demonstrated by blackberry extract was the result of the synergistic effects of various phytochemicals naturally present in blackberry fruits. In addition, the reduction in the secretion of pro-inflammatory cytokines makes blackberry an interesting source of bioactive compounds for the prevention and treatment of chronic inflammatory conditions [77,78] or COVID-19 disease [79], among others.

## 4. Conclusions

Blackberry fruits are a promising source of phytochemicals such as phenolic compounds and terpenoids. Among them, anthocyanins and the triterpenoid family of tormentic acid are outstanding for their abundance in the hydroalcoholic extract obtained in this study. To our knowledge, this is the first research in which terpenoids from the family of ursolic acid are described in blackberry fruits and are taken into account in the bioactivity potential of blackberry fruits. The in vitro biological activity assays of the extract have demonstrated its possible use for the prevention of oxidative stress and inflammation. In addition, blackberry extract may be a source of antimicrobials, molecules that might boost the immune system to fight infections, and that may also be involved in the prevention and treatment of different kinds of colorectal tumors. At this point, it is important to highlight the role of the intestinal microbiota in the modification of the molecular structures of bioactive compounds during the digestion process. Thus, future studies should be focused on the analytical characterization of the metabolites produced by the intestinal microbiota in a simulation of the digestion process of blackberry extract and the evaluation of the bioactivity of those metabolites to test if the molecular changes affect positively or negatively their bioactivity and bioavailability.

## Figures and Tables

**Table 1:** Identification and quantification of phenolic compounds present in blackberry extract.

Peak No.	Retention Time (Min)	m/z Exp.	m/z Calc.	Error (ppm)	Molecular Formula	Score	Proposed Compound	Quantification (μg/g d.w.)
Phenolic acids and derivatives								
3	0.433	333.0574	333.0610	−4.8	C_16_H_14_O_8_	93.93	Jaboticabin	27.68 ± 0.26
7	4.979	223.0598	223.0606	−3.6	C_11_H_12_O_5_	97.37	Sinapic acid	17.83 ± 0.12
9	5.476	385.1107	385.1135	−7.3	C_17_H_22_O_10_	92.67	Sinapic acid hexoside	13.35 ± 0.03
16	6.639	183.0249	183.0293	−2.4	C_8_H_8_O_5_	99.18	Methylgallic acid	19.18 ± 0.07
20	7.707	433.0406	433.0407	−0.2	C_19_H_14_O_12_	84.53	Ellagic acid-pentoside	27.98 ± 0.02
21	7.888	433.0412	433.0407	2.7	C_19_H_14_O_12_	92.90	Ellagic acid-pentoside isomer	25.62 ± 0.16
24	8.726	300.9994	300.9984	3.3	C_14_H_6_O_8_	100	Ellagic acid	19.46 ± 0.13
29	9.276	447.0560	447.0564	−0.9	C_20_H_16_O_12_	93.98	Ellagic acid 2-rhamnoside	20.89 ± 0.10
32	9.917	315.0119	315.0141	−7.0	C_15_H_8_O_8_	85.96	3-*O*-Methylellagic acid	10.41 ± 0.05
Flavonoids and derivatives								
6	4.367	463.0850	463.0877	−5.8	C_21_H_20_O_12_	95.62	Quercetin-*O*-hexoside	3.37 ± 0.09
10	5.487	577.1396	577.1405	−1.6	C_30_H_26_O_12_	85.36	B-type procyanidin dimer	7.57 ± 0.12
11	5.716	315.1226	315.1232	−1.9	C_18_H_20_O_5_	85.82	4-hydroxy-5,7,4′-trimethoxyflavan	5.89 ± 0.01
14	6.335	289.0714	289.0712	0.7	C_15_H_14_O_6_	87.91	Epicatechin	7.90 ± 0.03
15	6.517	577.1364	577.1346	3.1	C_30_H_26_O_12_	86.47	B-type procyanidin dimer isomer	7.94 ± 0.05
25	8.801	609.1475	609.1456	3.1	C_27_H_30_O_16_	99.80	Rutin	8.46 ± 0.15
26	9.046	463.0886	463.0877	1.9	C_21_H_20_O_12_	99.59	Quercetin 3-galactoside	5.63 ± 0.09
27	9.051	609.1460	609.1456	0.7	C_27_H_30_O_16_	99.55	Rutin isomer	5.50 ± 0.18
28	9.213	463.0854	463.0877	−5.0	C_21_H_20_O_12_	97.48	Quercetin-*O*-glucoside	3.96 ± 0.02
30	9.577	477.0656	477.0669	−2.7	C_21_H_18_O_13_	98.21	Quercetin 3-glucuronide	4.01 ± 0.01
34	10.199	505.1000	505.0982	3.6	C_23_H_22_O_13_	99.46	Quercetin-*O*-acetylhexoside	3.24 ± 0.11
Ellagitannins								
19	7.519	935.0790	935.0791	−0.1	C_41_H_28_O_26_	99.97	Casuarictin	21.02 ± 0.23
22	8.290	934.0745	934.0712	2.0	C_41_H_28_O_26_ (X2)	83.61	Sanguiin H6	25.99 ± 0.18
23	8.381	934.0761	934.0712	1.5	C_41_H_28_O_26_ (X2)	89.47	Sanguiin H6 isomer	35.09 ± 0.27
Lignans								
33	9.984	571.2175	571.2179	−0.7	C_30_H_36_O_11_	89.59	Kadsurarin	28.17 ± 0.14
35	10.509	571.2134	571.2179	−7.9	C_30_H_36_O_11_	85.74	Kadsurarin isomer	12.37 ± 0.06
36	10.740	341.1370	341.1389	−5.6	C_20_H_22_O_5_	99.89	Kadsurenin B	8.85 ± 0.10
Anthocyanins (MS+)								
12	5.859	449.1074	449.1084	−2.2	C_21_H_21_O_11_	99.91	Cyanidin-3-*O*-glucoside	1635.15 ± 13.24
17	6.691	595.1646	595.1663	−2.9	C_27_H_31_O_15_	92.30	Cyanidin-3-*O*-rutinoside	505.34 ± 5.37
18	7.268	419.0981	419.0919	0.7	C_20_H_19_O_10_	98.62	Cyanidin-3-*O*-arabinoside	145.05 ± 1.63
38	11.499	465.1038	465.1033	1.1	C_21_H_21_O_12_	96.29	Delphinidin-3-*O*-galactoside	32.46 ± 0.97
39	11.501	611.1598	611.1612	−2.3	C_27_H_31_O_16_	92.89	Cyanidin-3,5-*O*-diglucoside	132.11 ± 2.08
40	11.672	465.1042	465.1033	1.9	C_21_H_21_O_12_	99.40	Delphinidin-3-*O*-glucoside	63.56 ± 1.05
41	11.830	611.1587	611.1612	2.1	C_27_H_31_O_16_	91.56	Cyanidin-3-*O*-sophoroside	94.96 ± 1.58
43	13.572	465.1058	465.1033	5.4	C_21_H_21_O_12_	82.42	Delphinidin-3-*O*-galactoside	11.38 ± 0.46

**Table 2:** Terpenes and other polar compounds identified in blackberry fruit extract.

Peak No.	Retention Time (Min)	m/z Exp.	m/z Calc.	Error (ppm)	Molecular Formula	Score	Proposed Compound	%
Terpenoids								
5	4.023	443.1918	443.1917	0.2	C_21_H_32_O_10_	95.27	Ebuloside (iridoide)	0.54
8	5.189	517.2258	517.2285	−5.2	C_24_H_38_O_12_	84.32	Cinnamoside	0.63
12	6.117	347.1699	347.1706	−2	C_16_H_28_O_8_	99.96	Rhodioloside A	0.37
28	9.811	507.2219	507.2230	−2.2	C_26_H_36_O_10_	96.51	Scutalpin F	0.70
34	11.338	725.4130	725.4112	2.5	C_38_H_62_O_13_	99.64	Tropeoside B1	1.69
39	12.509	501.3188	501.3216	−5.6	C_30_H_46_O_6_	89.55	Dihydroxyurs-12-ene-23,28-dioic acid	0.64
41	13.760	487.3404	487.3423	−3.9	C_30_H_48_O_5_	89.45	Tormentic acid	0.25
42	13.760	709.4182	709.4163	2.7	C_38_H_62_O_12_	83.89	Elephanoside A	0.12
43	14.042	663.3893	663.3897	−0.6	C_40_H_56_O_8_	97.18	2a,3a-dihydroxy-24[(3-methoxy-4-hydroxy-trans-cinnamoyl)oxy]urs-12-en-28oic acid	2.02
44	14.119	519.3326	519.3322	0.8	C_30_H_48_O_7_	91.53	Dihydroxytormentic acid	0.35
45	14.203	533.3121	533.3114	1.3	C_30_H_46_O_8_	99.98	Tetrahydroxyurs-12-ene-23,28-dioic acid	1.02
46	14.324	533.3116	533.3114	0.4	C_30_H_46_O_8_	94.27	Tetrahydroxyurs-12-ene-23,28-dioic acid isomer a	1.83
47	14.401	533.3124	533.3114	1.9	C_30_H_46_O_8_	98.53	Tetrahydroxyurs-12-ene-23,28-dioic acid isomer b	0.30
48	14.673	519.3313	519.3322	−1.7	C_30_H_48_O_7_	90.55	Dihydroxytormentic acid isomer	0.63
49	14.736	517.3172	517.3165	1.4	C_30_H_46_O_7_	96.27	Corosin	0.92
50	15.157	517.3168	517.3165	0.6	C_30_H_46_O_7_	99.91	Corosin isomer a	12.06
51	15.454	503.3370	503.3373	−0.6	C_30_H_48_O_6_	98.98	Hydroxytormentic acid	15.05
52	15.553	503.3375	503.3373	0.4	C_30_H_48_O_6_	95.72	Hydroxytormentic acid isomer a	7.21
53	15.998	503.3371	503.3373	−0.4	C_30_H_48_O_6_	93.47	Hydroxytormentic acid isomer b	6.39
54	16.090	503.3366	503.3373	−1.4	C_30_H_48_O_6_	96.65	Hydroxytormentic acid isomer c	2.20
55	16.275	501.3214	501.3216	−0.4	C_30_H_46_O_6_	92.55	Dihydroxyurs-12-ene-23,28-dioic acid isomer a	4.41
56	16.367	501.3207	501.3216	−1.8	C_30_H_46_O_6_	95.06	Dihydroxyurs-12-ene-23,28-dioic acid isomer b	1.13
57	16.497	503.3361	503.3373	−1.2	C_30_H_48_O_6_	90.25	Hydroxytormentic acid isomer d	0.40
58	16.760	501.3202	501.3216	−2.8	C_30_H_46_O_6_	98.15	Dihydroxyurs-12-ene-23,28-dioic acid isomer c	2.85
59	16.875	487.3424	487.3423	0.2	C_30_H_48_O_5_	99.57	Tormentic acid isomer a	1.75
60	17.167	487.3422	487.3423	−0.2	C_30_H_48_O_5_	99.76	Tormentic acid isomer b	10.62
61	17.391	485.3272	485.3267	1	C_30_H_46_O_5_	99.12	Hydroxyurs-12-ene-23,28 dioic acid	6.26
62	17.497	517.3167	517.3165	0.4	C_30_H_46_O_7_	100	Corosin isomer b	6.85
63	17.668	471.3481	471.3474	1.5	C_30_H_48_O_4_	91.18	Rubitic acid	4.40
64	17.774	471.3472	471.3474	−0.4	C_30_H_48_O_4_	100	Rubitic acid isomer	3.84
65	12.979	721.3815	721.3799	3.4	C_38_H_58_O_13_	83.24	Suavissimoside F1	2.56
Other compounds								
1	0.414	353.0708	353.0720	−3.4	C_12_H_18_O_12_	99.21	6-O-(beta-D-glucopyranosyloxy)-L-ascorbic acid	
2	0.433	173.0069	173.0086	−9.8	C_6_H_6_O_6_	96.29	Dehydroascorbic acid	
4	1.240	219.0481	219.0505	−11	C_8_H_12_O_7_	96.56	Dimethyl citrate	
							Total terpenoid content (mg/g d.w.)	63.80

**Table 3:** Total antioxidants content, and antioxidant activity of blackberry extracts, quercetin and ascorbic acid (n = 3).

	Blackberry Extract	Quercetin	Ascorbic Acid
Folin–Ciocalteu (mg GAE/g d.w.)	31.1 ± 4.9	1019.7 ± 5.4	1260.2 ± 10.3
FRAP (μmol Fe^2+^/g d.w.)	637.8 ± 3.2	5927.2 ± 7.6	8639.7 ± 15.1
DPPH (IC_50_ μg d.w./mL)	97.1 ± 2.4	15.3 ± 0.1	9.6 ± 0.2
TEAC (μmol TE/g d.w.)	576.6 ± 8.3	1096.3 ± 8.7	1723.1 ± 10.4

**Table 4:** MBC/MFC of the blackberry extract.

	Strain Type	MBC/MFC, mg/mL
Gram-positive	*L. monocytogenes*	25
	*L. innocua*	25
	*S. aureus*	25
	*E. faecalis*	12.5
	*B. cereus*	12.5
Gram-negative	*S. enterica*	100
	*P. aeruginosa*	100
	*S. sonnei*	50
	*E. coli*	12.5
Fungi	*C. sake*	25
	*Z. bailii*	50
	*P. expansum*	100
	*A. niger*	100

**Table 5:** Antitumor and selectivity index of blackberry extract in HT-29, T-84 and SW-837 cell lines.

Cellular Line	IC_50_, mg/mL	Selectivity Index
HT-29 (Human grade II colorectal adenocarcinoma)	4.9 ± 0.2	22.1
T-84 (Human colorectal carcinoma)	5.9 ± 0.3	18.4
SW-837 (Human grade IV rectum adenocarcinoma)	5.9 ± 0.2	18.4

## Data Availability

Data are contained within the article.

## Supplementary Materials

The following supporting information can be downloaded at: https://www.mdpi.com/article/10.3390/foods12071505/s1, Table S1: Calibration ranges, curves, regression coefficients and LOD and LOQ of the standard molecules.

## References

[1] Verma R., Gangrade T., Punasiya R., Ghulaxe C. (2014). *Rubus fruticosus* (Blackberry) Use as an Herbal Medicine. Pharmacogn. Rev..

[2] Jara-Palacios M.J., Santisteban A., Gordillo B., Hernanz D., Heredia F.J., Escudero-Gilete M.L. (2019). Comparative Study of Red Berry Pomaces (Blueberry, Red Raspberry, Red Currant and Blackberry) as Source of Antioxidants and Pigments. Eur. Food Res. Technol..

[3] Bljajić K., Petlevski R., Vujić L., Čačić A., Šoštarić N., Jablan J., De Carvalho I.S., Končić M.Z. (2017). Chemical Composition, Antioxidant and α-Glucosidase-Inhibiting Activities of the Aqueous and Hydroethanolic Extracts of Vaccinium Myrtillus Leaves. Molecules.

[4] D’Angelo R.W.O., Gonçalves M.M., Fachi M.M., Vilhena R.d.O., Pontarolo R., Maluf D.F. (2020). UPLC–QToF-MS Characterization of Blackberry Extracts of Cultivars ‘Tupy’, ‘Guarani’, and ‘Xavante’: Development of Extract-Loaded Niosomes. Rev. Bras. Farmacogn..

[5] Dai J., Patel J.D., Mumper R.J. (2007). Characterization of Blackberry Extract and Its Antiproliferative and Anti-Inflammatory Properties. J. Med. Food.

[6] Hager T.J., Howard L.R., Liyanage R., Lay J.O., Prior R.L. (2008). Ellagitannin Composition of Blackberry as Determined by HPLC-ESI-MS and MALDI-TOF-MS. J. Agric. Food Chem..

[7] Oszmiański J., Nowicka P., Teleszko M., Wojdyło A., Cebulak T., Oklejewicz K. (2015). Analysis of Phenolic Compounds and Antioxidant Activity in Wild Blackberry Fruits. Int. J. Mol. Sci..

[8] Zia-Ul-Haq M., Riaz M., De Feo V., Jaafar H.Z.E., Moga M. (2014). *Rubus fruticosus* L.: Constituents, Biological Activities and Health Related Uses. Molecules.

[9] Ropiak H.M., Ramsay A., Mueller-Harvey I. (2016). Condensed Tannins in Extracts from European Medicinal Plants and Herbal Products. J. Pharm. Biomed. Anal..

[10] Albert C., Codină G.G., Héjja M., András C.D., Chetrariu A., Dabija A. (2022). Study of Antioxidant Activity of Garden Blackberries (*Rubus fruticosus* L.) Extracts Obtained with Different Extraction Solvents. Appl. Sci..

[11] D’Agostino M.F., Sicari V., Giuffrè A.M., Soria A.C. (2022). Blackberries (Rubus Ulmifolius Schott) from Calabria (Italy): A Comprehensive Characterisation. Eur. Food Res. Technol..

[12] Nile S.H., Park S.W. (2014). Edible Berries: Bioactive Components and Their Effect on Human Health. Nutrition.

[13] Dragana D.Č., Ranitovi A.S., Cvetkovi D.D., Markov S.L., Vincic M.N., Djilas S.M. (2017). Bioactivity of Blackberry (*Rubus fruticosus* L.) Pomace: Polyphenol Content, Radical Scavenging, Antimicrobial and Antitumor Activity. Acta Period. Technol..

[14] Halim M.A., Kanan K.A., Nahar T., Rahman M.J., Ahmed K.S., Hossain H., Mozumder N.H.M.R., Ahmed M. (2022). Metabolic Profiling of Phenolics of the Extracts from the Various Parts of Blackberry Plant (*Syzygium cumini* L.) and Their Antioxidant Activities. LWT.

[15] González O.A., Escamilla C., Danaher R.J., Dai J., Ebersole J.L., Mumper R.J., Miller C.S. (2013). Antibacterial Effects of Blackberry Extract Target Periodontopathogens. J. Periodontal Res..

[16] Mikulic-Petkovsek M., Slatnar A., Stampar F., Veberic R. (2012). HPLC-MS n Identification and Quantification of Flavonol Glycosides in 28 Wild and Cultivated Berry Species. Food Chem..

[17] Martin-Smith M., Khatoon T. (1963). Biological Activity of the Terpenoids and Their Derivatives. Fortschr. Arzneim..

[18] Yang W., Chen X., Li Y., Guo S., Wang Z., Yu X. (2020). Advances in Pharmacological Activities of Terpenoids. Nat. Prod. Commun..

[19] Folmer F., Basavaraju U., Jaspars M., Hold G., El-Omar E., Dicato M., Diederich M. (2014). Anticancer Effects of Bioactive Berry Compounds. Phytochem. Rev..

[20] Martín-García B., Aznar-Ramos M.J., Verardo V., Gómez-Caravaca A.M. (2022). Development of an Effective Sonotrode Based Extraction Technique for the Recovery of Phenolic Compounds with Antioxidant Activities in Cherimoya Leaves. Plants.

[21] Łukowski A., Jagiełło R., Robakowski P., Adamczyk D., Karolewski P. (2022). Adaptation of a Simple Method to Determine the Total Terpenoid Content in Needles of Coniferous Trees. Plant Sci..

[22] Ainsworth E.A., Gillespie K.M. (2007). Estimation of Total Phenolic Content and Other Oxidation Substrates in Plant Tissues Using Folin-Ciocalteu Reagent. Nat. Protoc..

[23] Re R., Pellegrini N., Proteggente A., Pannala A., Yang M., Rice-Evans C. (1999). Antioxidant Activity Applying an Improved ABTS Radical Cation Decolorization Assay. Free Radic. Biol. Med..

[24] Benzie I.F.F., Strain J.J. (1996). The Ferric Reducing Ability of Plasma (FRAP) as a Measure of “Antioxidant Power”: The FRAP Assay. Anal. Biochem..

[25] Brand-Williams W., Cuvelier M.E., Berset C. (1995). Use of a Free Radical Method to Evaluate Antioxidant Activity. LWT-Food Sci. Technol..

[26] Dayoub J.C., Ortiz F., Lõpez L.C., Venegas C., Del Pino-Zumaquero A., Roda O., Sánchez-Montesinos I., Acuña-Castroviejo D., Escames G. (2011). Synergism between Melatonin and Atorvastatin against Endothelial Cell Damage Induced by Lipopolysaccharide. J. Pineal Res..

[27] CLSI (2018). Methods for Dilution Antimicrobial Susceptibility Test for Bacteria That Grow Aerobically. https://clsi.org/standards/products/microbiology/documents/m07/.

[28] CLSI (2017). Reference Method for Broth Dilution Antifungal Susceptibility Testing of Filamentous Fungi. https://clsi.org/standards/products/microbiology/documents/m38/.

[29] Vichai V., Kirtikara K. (2016). Sulforhodamine B Colorimetric Assay for Cytotoxicity Screening. Nat. Protoc..

[30] Vezza T., Algieri F., Garrido-Mesa J., Utrilla M.P., Rodríguez-Cabezas M.E., Baños A., Guillamón E., García F., Rodríguez-Nogales A., Gálvez J. (2019). The Immunomodulatory Properties of Propyl-Propane Thiosulfonate Contribute to Its Intestinal Anti-Inflammatory Effect in Experimental Colitis. Mol. Nutr. Food Res..

[31] Connor A.M., Finn C.E., McGhie T.K., Alspach P.A. (2005). Genetic and Environmental Variation in Anthocyanins and Their Relationship to Antioxidant Activity in Blackberry and Hybridberry Cultivars. J. Am. Soc. Hortic. Sci..

[32] Mullen W., Larcombe S., Arnold K., Welchman H., Crozier A. (2010). Use of Accurate Mass Full Scan Mass Spectrometry for the Analysis of Anthocyanins in Berries and Berry-Fed Tissues. J. Agric. Food Chem..

[33] Santos S.S.d., Magalhães F.d.S., Paraíso C.M., Ogawa C.Y.L., Sato F., Santos Junior O.d.O., Visentainer J.V., Madrona G.S., Reis M.H.M. (2022). Enhanced Conditions for Anthocyanin Extraction from Blackberry Pomace under Ultrasound Irradiation. J. Food Process Eng..

[34] Zhao D.K., Shi Y.N., Petrova V., Yue G.G.L., Negrin A., Wu S.B., D’Armiento J.M., Lau C.B.S., Kennelly E.J. (2019). Jaboticabin and Related Polyphenols from Jaboticaba (*Myrciaria cauliflora*) with Anti-Inflammatory Activity for Chronic Obstructive Pulmonary Disease. J. Agric. Food Chem..

[35] Čanadanović-Brunet J., Tumbas Šaponjac V., Stajčić S., Ćetković G., Čanadanović V., Ćebović T., Vulić J. (2019). Polyphenolic Composition, Antiradical and Hepatoprotective Activities of Bilberry and Blackberry Pomace Extracts. J. Berry Res..

[36] Moraes D.P., Machado M.L., Farias C.A.A., Barin J.S., Zabot G.L., Lozano-Sánchez J., Ferreira D.F., Vizzotto M., Leyva-Jimenez F.J., Da Silveira T.L. (2020). Effect of Microwave Hydrodiffusion and Gravity on the Extraction of Phenolic Compounds and Antioxidant Properties of Blackberries (*Rubus* spp.): Scale-Up Extraction. Food Bioprocess Technol..

[37] Jin Cho M., Howard L.R., Prior R.L., Clark J.R. (2005). Flavonol Glycosides and Antioxidant Capacity of Various Blackberry and Blueberry Genotypes Determined by High-Performance Liquid Chromatography/Mass Spectrometry. J. Sci. Food Agric..

[38] Krstić Đ.D., Ristivojević P.M., Gašić U.M., Lazović M., Fotirić Akšić M.M., Milivojević J., Morlock G.E., Milojković-Opsenica D.M., Trifković J. (2023). Authenticity Assessment of Cultivated Berries via Phenolic Profiles of Seeds. Food Chem..

[39] Wang W., Liu J., Liu R., Xu Z., Yang M., Wang W., Liu P., Sabia G., Wang X., Guo D. (2006). Four New Lignans from the Stems of Kadsura Heteroclita. Planta Med..

[40] Shokrzadeh M., Saeedi Saravi S.S. (2010). The Chemistry, Pharmacology and Clinical Properties of Sambucus Ebulus: A Review. J. Med. Plants Res..

[41] Shiraga Y., Okano K., Akira T., Fukaya C., Yokoyama K., Tanaka S., Fukui H., Tabata M. (1988). Structures of Potent Antiulcerogenic Compounds from Cinnamomum Cassia. Tetrahedron.

[42] Zakharenko A.M., Razgonova M.P., Pikula K.S., Golokhvast K.S. (2021). Simultaneous Determination of 78 Compounds of Rhodiola Rosea Extract by Supercritical CO2-Extraction and HPLC-ESI-MS/MS Spectrometry. Biochem. Res. Int..

[43] Malakov P.Y., Papanov G.Y. (1998). Neo-Clerodane Diterpenoids from Scutellaria Alpina. Phytochemistry.

[44] Corea G., Fattorusso E., Lanzotti V., Capasso R., Izzo A.A. (2005). Antispasmodic Saponins from Bulbs of Red Onion, *Allium cepa* L. Var. Tropea. J. Agric. Food Chem..

[45] Gradillas A., Martínez-Alcázar M.P., Gutiérrez E., Ramos-Solano B., García A. (2019). A Novel Strategy for Rapid Screening of the Complex Triterpene Saponin Mixture Present in the Methanolic Extract of Blackberry Leaves (Rubus Cv. Loch Ness) by UHPLC/QTOF-MS. J. Pharm. Biomed. Anal..

[46] Youn H.J., Kim K.B., Han H.S., An I.S., Ahn K.J. (2017). 23-Hydroxytormentic Acid Protects Human Dermal Fibroblasts by Attenuating UVA-Induced Oxidative Stress. Photodermatol. Photoimmunol. Photomed..

[47] Wang Y., Liu F., Liu P. (2021). 23-Hydroxytormentic Acid Reduces Cerebral Ischemia/Reperfusion Damage in Rats through Anti-Apoptotic, Antioxidant, and Anti-Inflammatory Mechanisms. Naunyn. Schmiedebergs. Arch. Pharmacol..

[48] Olech M., Ziemichód W., Nowacka-jechalke N. (2021). The Occurrence and Biological Activity of Tormentic Acid—A Review. Molecules.

[49] Jäger S., Trojan H., Kopp T., Laszczyk M.N., Scheffler A. (2009). Pentacyclic Triterpene Distribution in Various Plants—Rich Sources for a New Group of Multi-Potent Plant Extracts. Molecules.

[50] Georgé S., Brat P., Alter P., Amiot M.J. (2005). Rapid Determination of Polyphenols and Vitamin C in Plant-Derived Products. J. Agric. Food Chem..

[51] Lamuela-Raventós R.M. (2017). Folin-Ciocalteu Method for the Measurement of Total Phenolic Content and Antioxidant Capacity. Meas. Antioxid. Act. Capacit. Recent Trends Appl..

[52] Jazić M.R., Vulić J.J., Kukrić Z.Z., Topalić-Trivunović L.N., Savić A.V. (2018). Chemical Composition, Biological Potentials and Antimicrobial Activity of Wild and Cultivated Blackberries. Acta Period. Technol..

[53] Wajs-Bonikowska A., Stobiecka A., Bonikowski R., Krajewska A., Sikora M., Kula J. (2017). A Comparative Study on Composition and Antioxidant Activities of Supercritical Carbon Dioxide, Hexane and Ethanol Extracts from Blackberry (*Rubus fruticosus*) Growing in Poland. J. Sci. Food Agric..

[54] Zorzi M., Gai F., Medana C., Aigotti R., Morello S., Peiretti P.G. (2020). Small Berries. Foods.

[55] Graßmann J. (2005). Terpenoids as Plant Antioxidants. Vitam. Horm..

[56] Porte S., Joshi V., Shah K., Chauhan N.S. (2022). Plants’ Steroidal Saponins—A Review on Its Pharmacology Properties and Analytical Techniques. World J. Tradit. Chinese Med..

[57] Higashi-Okai K., Ishikawa A., Yasumoto S., Okai Y. (2009). Potent Antioxidant and Radical-Scavenging Activity of Chenpi—Compensatory and Cooperative Actions of Ascorbic Acid and Citric Acid. J. UOEH.

[58] Li J., Pettinato M., Campardelli R., De Marco I., Perego P. (2022). High-Pressure Technologies for the Recovery of Bioactive Molecules from Agro-Industrial Waste. Appl. Sci..

[59] Mitra S., Tareq A.M., Das R., Emran T.B., Nainu F., Chakraborty A.J., Ahmad I., Tallei T.E., Idris A.M., Simal-Gandara J. (2022). Polyphenols: A First Evidence in the Synergism and Bioactivities. Food Rev. Int..

[60] Wrońska N., Szlaur M., Zawadzka K., Lisowska K. (2022). The Synergistic Effect of Triterpenoids and Flavonoids—New Approaches for Treating Bacterial Infections?. Molecules.

[61] Sotler R., Poljšak B., Dahmane R., Jukić T., Pavan Jukić D., Rotim C., Trebše P., Starc A. (2019). Prooxidant Activities of Antioxidants and Their Impact on Health. Acta Clin. Croat..

[62] Azofeifa G., Quesada S., Pérez A.M., Vaillant F., Michel A. (2015). Pasteurization of Blackberry Juice Preserves Polyphenol-Dependent Inhibition for Lipid Peroxidation and Intracellular Radicals. J. Food Compos. Anal..

[63] Hassan H.A., Abdel-Aziz A.F. (2010). Evaluation of Free Radical-Scavenging and Anti-Oxidant Properties of Black Berry against Fluoride Toxicity in Rats. Food Chem. Toxicol..

[64] Cho B.O., Ryu H.W., Jin C.H., Choi D.S., Kang S.Y., Kim D.S., Byun M.W., Jeong I.Y. (2011). Blackberry Extract Attenuates Oxidative Stress through Up-Regulation of Nrf2-Dependent Antioxidant Enzymes in Carbon Tetrachloride-Treated Rats. J. Agric. Food Chem..

[65] Radovanović B.C., Andelković A.S.M., Radovanović A.B., Andelković M.Z. (2013). Antioxidant and Antimicrobial Activity of Polyphenol Extracts from Wild Berry Fruits Grown in Southeast Serbia. Trop. J. Pharm. Res..

[66] Katerere D.R., Gray A.I., Nash R.J., Waigh R.D. (2003). Antimicrobial Activity of Pentacyclic Triterpenes Isolated from African Combretaceae. Phytochemistry.

[67] Wang C.M., Chen H.T., Wu Z.Y., Jhan Y.L., Shyu C.L., Chou C.H. (2016). Antibacterial and Synergistic Activity of Pentacyclic Triterpenoids Isolated from Alstonia Scholaris. Molecules.

[68] Baysal G., Olcay H.S., Keresteci B., Özpinar H. (2022). The Antioxidant and Antibacterial Properties of Chitosan Encapsulated with the Bee Pollen and the Apple Cider Vinegar. J. Biomater. Sci. Polym. Ed..

[69] Tian Y., Puganen A., Alakomi H.L., Uusitupa A., Saarela M., Yang B. (2018). Antioxidative and Antibacterial Activities of Aqueous Ethanol Extracts of Berries, Leaves, and Branches of Berry Plants. Food Res. Int..

[70] Garcia-Rubio R., de Oliveira H.C., Rivera J., Trevijano-Contador N. (2020). The Fungal Cell Wall: Candida, Cryptococcus, and Aspergillus Species. Front. Microbiol..

[71] Seeram N.P., Adams L.S., Zhang Y., Lee R., Sand D., Scheuller H.S., Heber D. (2006). Blackberry, Black Raspberry, Blueberry, Cranberry, Red Raspberry, and Strawberry Extracts Inhibit Growth and Stimulate Apoptosis of Human Cancer Cells in Vitro. J. Agric. Food Chem..

[72] Jazić M., Kukrić Z., Vulić J., Četojević-Simin D. (2019). Polyphenolic Composition, Antioxidant and Antiproliferative Effects of Wild and Cultivated Blackberries (*Rubus fruticosus* L.) Pomace. Int. J. Food Sci. Technol..

[73] Rodrigues C.A., Nicácio A.E., Boeing J.S., Garcia F.P., Nakamura C.V., Visentainer J.V., Maldaner L. (2020). Rapid Extraction Method Followed by a D-SPE Clean-up Step for Determination of Phenolic Composition and Antioxidant and Antiproliferative Activities from Berry Fruits. Food Chem..

[74] Gil-Sánchez I., Cueva C., Tamargo A., Quintela J.C., de la Fuente E., Walker A.W., Moreno-Arribas M.V., Bartolomé B. (2020). Application of the Dynamic Gastrointestinal Simulator (Simgi^®^) to Assess the Impact of Probiotic Supplementation in the Metabolism of Grape Polyphenols. Food Res. Int..

[75] Lopez-Corona A.V., Valencia-Espinosa I., González-Sánchez F.A., Sánchez-López A.L., Garcia-Amezquita L.E., Garcia-Varela R. (2022). Antioxidant, Anti-Inflammatory and Cytotoxic Activity of Phenolic Compound Family Extracted from Raspberries (*Rubus idaeus*): A General Review. Antioxidants.

[76] Tatipamula V.B., Kukavica B. (2021). Phenolic Compounds as Antidiabetic, Anti-Inflammatory, and Anticancer Agents and Improvement of Their Bioavailability by Liposomes. Cell Biochem. Funct..

[77] Yahfoufi N., Alsadi N., Jambi M., Matar C. (2018). The Immunomodulatory and Anti-Inflammatory Role of Polyphenols. Nutrients.

[78] Lail H.L., Feresin R.G., Hicks D., Stone B., Price E., Wanders D. (2021). Berries as a Treatment for Obesity-Induced Inflammation: Evidence from Preclinical Models. Nutrients.

[79] Iqhrammullah M., Rizki D.R., Purnama A., Duta T.F. (2023). Antiviral Molecular Targets of Essential Oils against SARS-CoV-2: A Systematic Review. Sci. Pharm..

