# Supplementary material for: Phytochemicals Determination, and Antioxidant, Antimicrobial, Anti-Inflammatory and Anticancer Activities of Blackberry Fruits
Source: Foods. 2023 Apr 3;12(7):1505. doi: 10.3390/foods12071505 (PMC10094647; doi:10.3390/foods12071505)
Supplement: Supplementary file 1 [file foods-12-01505-s001.zip › foods-2285731-supplementary.pdf]

Table S1. Calibration ranges, curves, regression coefficients and LOD and LOQ of the standard molecules

Standards	Calibration Ranges (µg/mL)	Calibration Curves (µg/mL)	R2	LOD (µg/mL)	LOQ (µg/mL)
Chlorogenic acid	5.78-185.00	y=49.157x-227.23	0.9918	0.03	0.09
Ferulic acid	5.31-170.00	y=22.075x-65	0.9994	0.05	0.16
Rutin	5.15-165.00	y=549.78x+1547.8	0.9981	0.04	0.12
Catechin	4.43-140.00	y=434.59x-3192.2	0.9977	0.04	0.14
Quercetin	5.31-170.00	y=101.89x-461.56	0.9967	0.03	0.10
Cyanidin-glucoside	2.85-200.00	y=51.349x+1.09	0.9989	0.006	0.02
